# Using phenotype microarrays in the assessment of the antibiotic susceptibility profile of bacteria isolated from wastewater in on-site treatment facilities

**DOI:** 10.1007/s12223-017-0516-9

**Published:** 2017-04-27

**Authors:** Łukasz Jałowiecki, Joanna Chojniak, Elmar Dorgeloh, Berta Hegedusova, Helene Ejhed, Jörgen Magnér, Grażyna Płaza

**Affiliations:** 1 Department of Environmental Microbiology, Institute for Ecology of Industrial Areas, Katowice, Poland; 2 Development and Assessment Institute in Waste Water Technology, RWTH Aachen University, Aachen, Germany; 3 Natural Resources and Environmental Effects, IVL Swedish Environmental Research Institute, Stockholm, Sweden

## Abstract

The scope of the study was to apply Phenotype Biolog MicroArray (PM) technology to test the antibiotic sensitivity of the bacterial strains isolated from on-site wastewater treatment facilities. In the first step of the study, the percentage values of resistant bacteria from total heterotrophic bacteria growing on solid media supplemented with various antibiotics were determined. In the untreated wastewater, the average shares of kanamycin-, streptomycin-, and tetracycline-resistant bacteria were 53, 56, and 42%, respectively. Meanwhile, the shares of kanamycin-, streptomycin-, and tetracycline-resistant bacteria in the treated wastewater were 39, 33, and 29%, respectively. To evaluate the antibiotic susceptibility of the bacteria present in the wastewater, using the phenotype microarrays (PMs), the most common isolates from the treated wastewater were chosen: *Serratia marcescens ss marcescens*, *Pseudomonas fluorescens*, *Stenotrophomonas maltophilia*, *Stenotrophomonas rhizophila*, *Microbacterium flavescens*, *Alcaligenes faecalis ss faecalis*, *Flavobacterium hydatis*, *Variovorax paradoxus*, *Acinetobacter johnsonii*, and *Aeromonas bestiarum.* The strains were classified as multi-antibiotic-resistant bacteria. Most of them were resistant to more than 30 antibiotics from various chemical classes. Phenotype microarrays could be successfully used as an additional tool for evaluation of the multi-antibiotic resistance of environmental bacteria and in preliminary determination of the range of inhibition concentration.

## Introduction

Antibiotic resistance was identified by the World Health Organization as a major problem in terms of the environment and human and animal health (WHO 2014). Antibiotics get into soil and water, mainly through wastewater treatment plant effluents, leakage from waste storage containers, agricultural waste, and application of biosolids to fields (Dias et al. 2015).

Antibiotic resistance is a highly selectable phenotype and can be detected using the traditional microbiological methods (culture-based approaches) and modern techniques based on nucleic acid approaches. The conventional methods for susceptibility testing require the isolation of the bacteria from the environmental samples and culturing on the appropriate media that contain antibiotic(s) (Dias et al. 2015). The most popular are growth inhibition assays performed in broth or by an agar disc diffusion method. In a dilution-based growth inhibition assay, the minimum inhibitory concentration (MIC) of an antibiotic can be calculated for each bacterial isolate, and the bacteria are classified as being susceptible or resistant to the antibiotic.

Nucleic acid-based approaches offer rapid and sensitive methods to detect the resistance genes and play a critical role in the elucidation of resistance mechanisms, and they are particularly useful for slow-growing or non-culturable microorganisms and for the detection of point mutations or certain genotypes. During the last decade, nucleic acid-based detection systems have expanded tremendously and are becoming more accessible for clinical studies (Bergeron 2000; Fluit et al. 2001). Polymerase chain reaction (PCR) is one of the most commonly used molecular techniques for detecting certain DNA sequences of interest. Frickmann et al. (2014) reviewed the antimicrobial susceptibility testing methods that have been developed recently. They include classical agglutination assays, molecular testing methods, for example, qPCR, DNA microarrays, Luminex xMAP assays and next generation sequencing, fluorescence in situ hybridization (FISH), and mass spectrometry-based methods, for example, phyloproteomics, assays using stable isotope labeling of amino acids, mass spectrometric beta-lactamase assays, PCR/electrospray ionization mass spectrometry (PCR/ESI MS), minisequencing, and mass spectrometry-based comparative sequence analysis (MSCSA). A few microarrays have been developed for identification of antibiotic resistance genes (Call et al. 2003; Monecke et al. 2003; Holzman 2003; Perreten et al. 2005). While Schmieder and Edwards (2012) described the metagenomic as modern approaches that overcome the limitations of methods based on culturing or amplification.

In the context of the methods presented above, the purpose of this study was to detect antibiotic profiles of environmental bacteria by the phenotype microarrays (PMs).

## Materials and methods

### Description of on-site wastewater treatment facilities and sampling

The wastewater samples were collected from three different biological facilities of on-site wastewater treatment named A, B, and C. Facilities A and B are based on biofilm technology on carrier materials in which microorganisms degrade organic contaminants in the wastewater while being attached to different carrier materials and forming a biofilm. Facility C uses a combination of the activated sludge technology and biofilm technology. Detailed description of the facilities is presented in (Jałowiecki et al. 2016). The following samples were collected: influent, effluent, and sludge (liquid from the bioreactor) from facility A, influent, effluent, and sludge (rock wool pieces) from facility B, and influent, effluent, and sludge (carrier media, liquid from the bioreactor) from facility C. All grab (or catch) samples were collected manually by trained personnel. A 1000 mL volume was chosen for every sample and 500 g of for every carrier media. The sample material was placed immediately in a plastic, screw-capped container, and the containers were placed in a shipping box. Appropriate sample storage conditions were ensured together with the shortest transport and storage time. All the samples collected were stored in the sterile polypropylene (PP) bottles at 4 °C for microbiological analysis within 24 h from the sampling. The samples were evaluated in three replicates.

### Isolation and identification of bacterial strains

Culturable bacteria were evaluated in series with a tenfold dilutions of the liquid sample, i.e., 1 mL of the liquid sample was dispersed in 9 mL of sterilized physiological solution (0.85% NaCl) by shaking for 2 min. One milliliter of aliquots of the dilutions (10^−3^–10^−6^) was pipetted onto plates. Then a pour-plate method was used for evaluation of the number of bacteria. Three replicates were made per dilution. Bacteria were grown on SMA medium (peptone—8 g/L, yeast extract—2.5 g/L, glucose—1 g/L, agar—20 g/L, pH 7.0 ± 0.2; Standard Methods Agar, BioMerieux) supplemented with the following singular antibiotics: kanamycin (16 mg/L), streptomycin (30 mg/L), and tetracycline (16 mg/L) and in following combinations: kanamycin + tetracycline, tetracycline + penicillin (30 mg/L) + streptomycin and kanamycin + penicillin + streptomycin. The Petri dishes were incubated at 30 °C for 48–72 h. These antibiotics’ concentrations were determined in the previous experiments (Jałowiecki et al. 2016). Bacterial colonies which appeared on the media were counted and expressed in colony forming units (CFU), then the population data were transformed to log CFU, and percentage of antibiotic-resistant bacteria relative to the control without the antibiotics (heterotrophic number of bacteria) was calculated. Based on their different morphological characteristics (e.g., color, surface, the margin of the colony), the bacteria were chosen for further studies. The bacteria were picked up and purified to obtain a single colony. Currently, the bacterial isolates are stored in tryptic soy broth with 20% glycerol at −20 °C. In total amount, around 100 bacterial isolates from the samples were selected for the identification.

The identification of selected bacteria was performed by a new GEN III MicroPlate™ test panel of the Biolog system. The GEN III MicroPlates™ enable testing of Gram-negative and Gram-positive bacteria in the same test panel. The test panel contains 71 carbon sources and 23 chemical sensitivity assays. GEN III analyzes the ability of the cell to metabolize all major classes of compounds, in addition to determining other important physiological properties such as pH, salt and lactic acid tolerance, reducing power, and chemical sensitivity. All the reagents used in the experiment were originally obtained from Biolog, Inc. (Hayward, CA, USA). The bacterial suspensions for the identification test were prepared as recommended by the manufacturer. The plates were incubated at 30 °C in an Omnilog Reader/Incubater (Biolog). After incubation, the phenotypic fingerprint of purple wells was compared to Biolog’s extensive species library. If a match was found, a species level identification of the isolates could be made.

### Evaluation of antibiotic resistance of isolated strains by BIOLOG^TM^ PM microplates

PM panels are 96 well microplates containing different substrates in each well. PM11 and PM12 assays were used to determine the antibiotic resistance of the bacteria. In addition to a unique substrate (antibiotics), each well of the panels also contains the needed minimal medium components and specific dye. The arrays provide the identification of resistance to 41 antibiotics belonging to the ten different chemical classes, e.g., aminoglycosides, β-lactams, lincosamides, synthetic antibiotics, glycopeptides, tetracyclines, amphenicols, macrolides, sulfonamides, and rifamycins. Each antibiotic sensitivity assay includes four increasing concentrations of the test antibiotic. The strains were considered as resistant or insensitive to an antibiotic when there was a 100% increase in growth in at least two out of these four concentrations. The PM technology is based on culturing.

The most common ten bacteria from the treated wastewater were chosen for this analysis. The strains were grown overnight at 30 °C on SMA (Standard Methods Agar, BioMérieux) medium, and then cells were picked up with a sterile cotton swab and transferred into a sterile capped tube containing 20 mL of the inoculation fluid (IF-0, Biolog Inc.). The cell concentration was adjusted to 81% transmittance on the Biolog turbidimeter. After that, the PM11 and PM12 plates were inoculated with the cell suspension (100 μL per well) and incubated at 30 °C during 48 h in the Omnilog Incubater/Reader (Biolog Inc., Hayward, USA). The changes of color in the wells were measured every 15 min provided both amplification and quantitation of the phenotype. Analysis was carried out using OmniLog® phenotype microarray software v 1.2 to determine the phenotypic differences. The data were collected using OmniLog® MicroArray^TM^ Data Collection Software Release 1.2 (Biolog Inc.), which generated a tetrazolium salt color development as a function of time. The growth of bacteria was noted for the OmniLog values greater than 200.

## Results and discussion

**Fig. 1:**
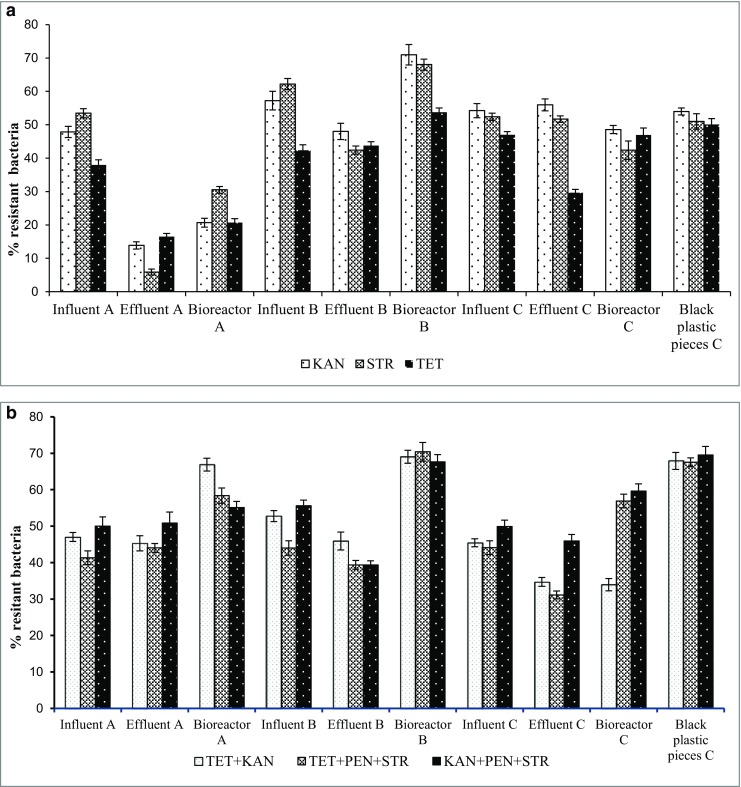
The percentage values of resistant bacteria in the tested samples from the three on-site wastewater treatment facilities. *Error bars* indicate standard deviation for replicates from single sampling events. **a** The percentage values of bacteria resistant to antibiotics alone: KAN, STR, and TET. **b** The percentage values of bacteria resistant to the antibiotic mixture: TET + KAN, TET + PEN + STR, KAN + PEN + STR. Abbreviations: *KAN* kanamycin, *STR* streptomycin, *TET* tetracycline, *PEN* penicillin

In order to compare the results from this study with those carried out by other researchers, the percentages of antibiotic-resistant bacteria from the total heterotrophic bacteria growing on solid media supplemented with antibiotics were calculated. The results are presented in Fig. 1. The percentage values of kanamycin-, streptomycin-, and tetracycline-resistant bacteria in the influent raw wastewaters were 53, 56, and 42%, respectively. Meanwhile, the percentage values of kanamycin-, streptomycin-, and tetracycline-resistant bacteria in the effluent treated wastewaters were 39, 33, and 29%, respectively. A similar relation was observed for the mixture of the antibiotics, e.g., tetracycline + kanamycin-, tetracycline + penicillin + streptomycin-, kanamycin + penicillin + streptomycin-resistant, the percentage values of bacteria in the influents were 48, 43, and 52%, respectively. However, the percentage values of tetracycline + kanamycin-, tetracycline + penicilin + streptomycin-, kanamycin + penicilin + streptomycin-resistant bacteria in the effluents were 42, 38, and 45%, respectively. The highest percentage values of antibiotic-resistant bacteria were in the biofilm carrier samples in facility B, e.g., rock wool which was used as a trickling biofilter media in facility B and in the sample from black plastic pieces obtained from facility C, e.g., small, fluidized units of carrier media providing a high active surface for growing of microorganisms. The scientific literature on the antibiotic resistance of bacterial communities from small wastewater treatment facilities is limited. Huang et al. (2012) evaluated the level of antibiotic tolerance of heterotrophic bacteria and investigated the distribution of bacterial resistance to six different antibiotics (penicillin, ampicillin, cephalothin, chloramphenicol, tetracycline, rifampicin) in the secondary effluent of the wastewater treatment plant to provide useful information on antibiotic-resistant bacteria and suspected risk of antibiotic resistance to natural waters. The average percentages of penicillin-, ampicillin-, cephalothin-, chloramphenicol-, tetracycline-, and rifampicin-resistant heterotrophic bacteria in the effluents were 63, 47, 55, 69, 2.6, and 11%, respectively. The number of tetracycline- and rifampicin-resistant bacteria was found to be much lower than the other four. When comparing the results obtained in this study on on-site wastewater treatment facilities with the data from the literature on centralized wastewater plants, the percentages of antibiotic-resistant heterotrophic bacteria occurred at the similar levels in the both wastewater treatment systems. The effluents from wastewater treatment plants from both wastewater treatment plants and small (domestic) systems could be a source of antibiotic-resistant bacteria and antibiotic resistance genes spread into the natural environment and also could transfer the antibiotic resistance to more pathogenic or non-antibiotic-resistant bacteria.

There is also limited research on the characterization of antibiotic-resistant bacterial communities in environmental samples, such as raw and treated wastewater from on-site wastewater treatment facilities (Novo and Manaia 2010; Huang et al. 2012). Most studies on antibiotic-resistant bacteria worked on single isolates and tested the antibiotic resistance of these single strains, mainly pathogens by antibiotic susceptibility testing such as the disc diffusion method (Silva et al. 2006; Huang et al. 2012; Boczek et al. 2007; Silva et al. 2007; Baquero et al. 2008; Pignato et al. 2009; Zhang et al. 2009; Wang et al. 2013; Płaza et al. 2013; Adegoke and Okoh 2014).

**Fig. 2:**
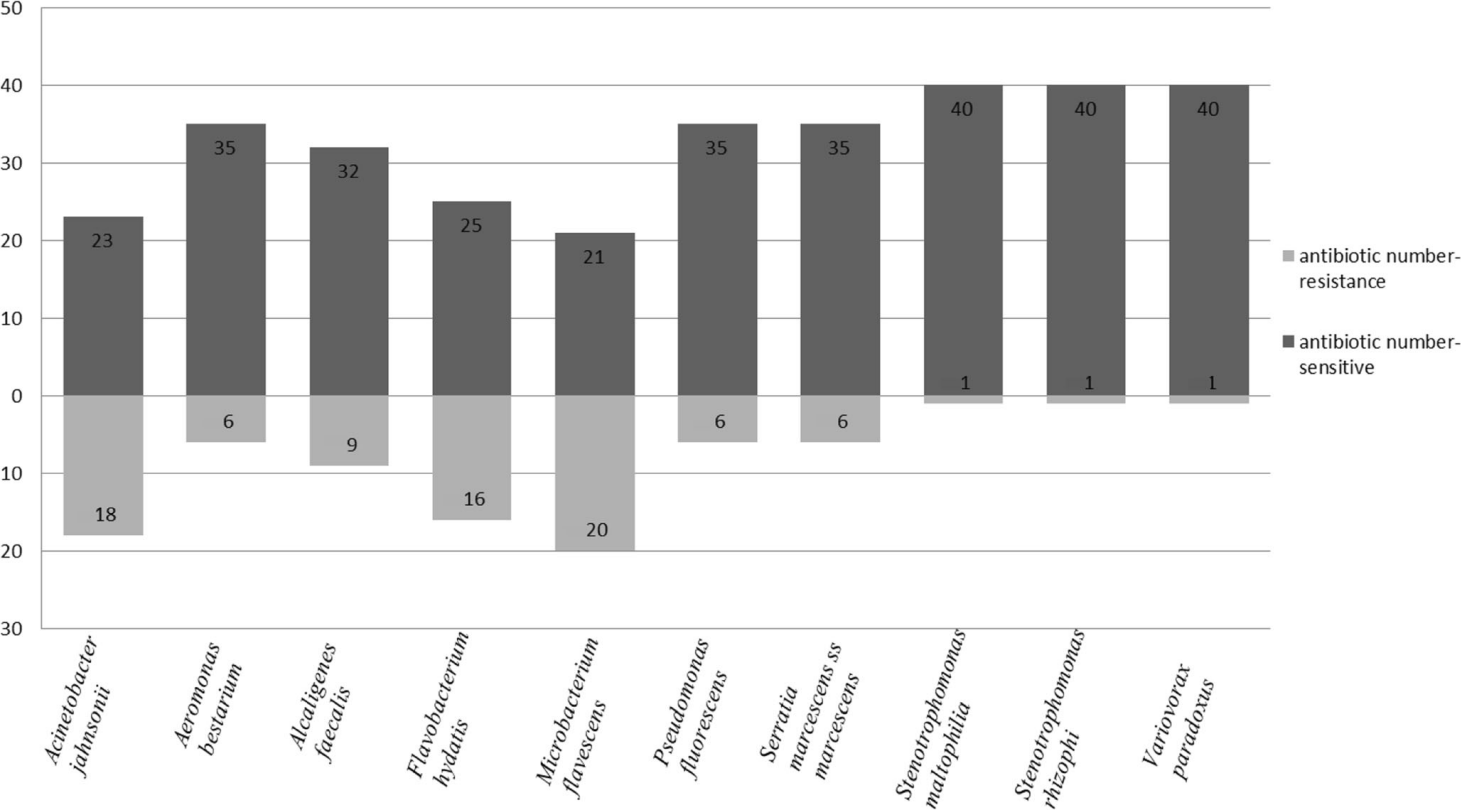
Number of antibiotics to which selected strains are resistant or sensitive

**Fig. 3:**
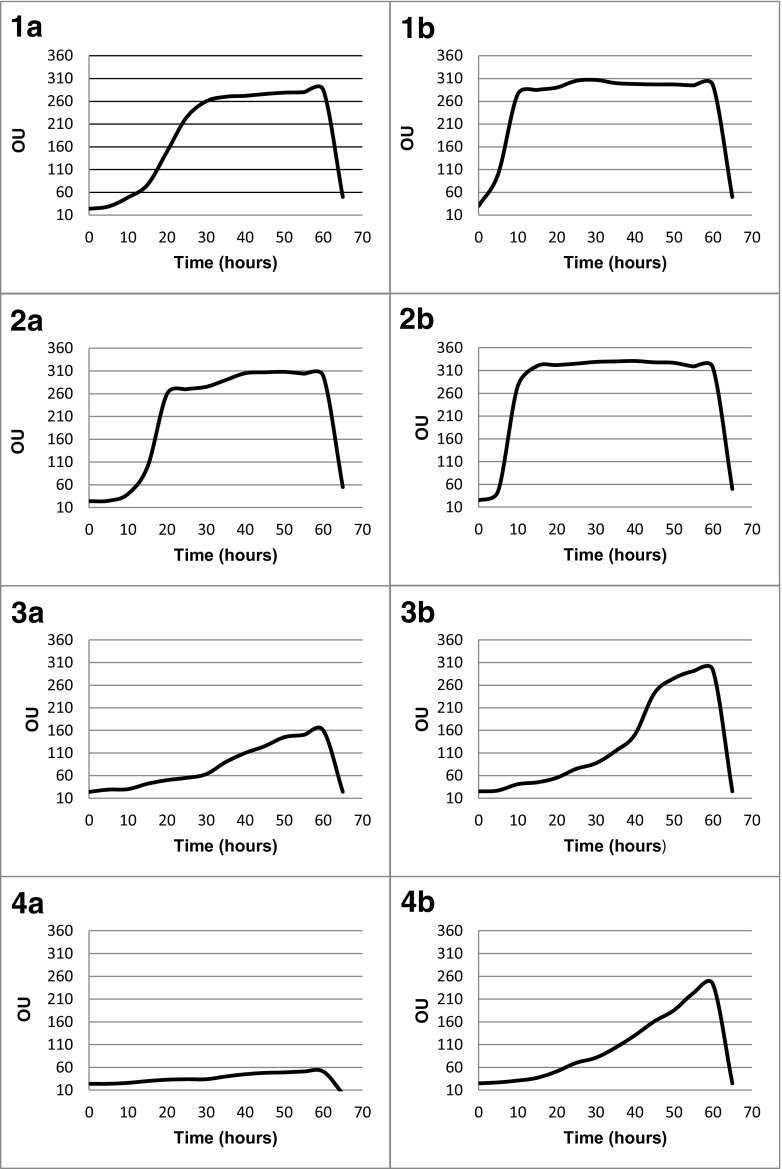
The scheme of antibiotic sensitivity profile obtained by using the PMs for two antibiotics: enoxacin (*1A*–*4A*) and tetracycline (*1B*–*4B*). Each antibiotic is presented at four concentrations. The concentrations of enoxacin and tetracycline in the following wells are increased from 0.04 to 4.00 μg/mL and from 0.08 to 8.00 μg/mL, respectively. Antibiotic sensitivity profile displayed in the form of kinetic graphs of the bacteria growth (time versus absorbance). The growth curves (in the individual four wells) show the time course (*horizontal axis*) of the amount of purple color formed from tetrazolium dye reduction (*vertical axis*) determined by the Omnilog unit (OU). The graphs were generated by the OmniLog® MicroArray^TM^ Data Collection Software Release 1.2 (Biolog Inc.)

PMs is a high-throughput technology for characterization and monitoring the microbial cellular phenotypes. It provides a set of nearly 2000 culture conditions including 200 carbon and 400 nitrogen sources, 100 phosphorous and sulfur sources, 100 nutrient supplements, and a range pH, gradients of osmolytes, and 240 toxic chemicals at four different concentrations including antibiotics (Adegoke and Okoh 2014; Bochner et al. 2001; Bochner 2009; Bochner et al. 2008). In our previous study, the following phenotype microarrays were used: GEN III plates, new test panel for identification of both Gram-negative and Gram-positive bacteria, EcoPlates microarray for evaluation of functional diversity of microbial communities, and PMs for characterization of the selected bacterial strains isolated from the water contaminated by the phenolic compounds (Chojniak et al. 2015). Therefore, our experiment attempted to evaluate the PM11 and PM12 for antibiotic sensitivity analysis of ten environmental strains: *Serratia marcescens ss marcescens*, *Pseudomonas fluorescens*, *Stenotrophomonas maltophilia*, *Stenotrophomonas rhizophila*, *Microbacterium flavescens*, *Alcaligenes faecalis ssp. faecalis*, *Flavobacterium hydatis*, *Variovorax paradoxus*, *Acinetobacter johnsonii*, and *Aeromonas bestiarum*. PM analysis showed resistance of these strains to 41 antibiotics belonging to the ten different chemical classes, e.g., aminoglycosides, β-lactams, lincosamides, synthetic antibiotics, glycopeptides, tetracyclines, amphenicols, macrolides, sulfonamides, and rifamycins. The results obtained are presented in Table 1. All the tested strains showed the growth in the presence of many antibiotics (Fig. 2). Two species belonging to the genus *Stenotrophomonas* (*S. maltophila* and *S. rhizophila*) and *Variovorax paradoxus* were resistant to 40 antibiotics. The rest of bacteria were resistant from 21 to 35 antibiotics. All tested strains could be named as multi-resistant bacteria, e.g., they are resistant to several antibiotics belonging to the ten different classes. Because each antibiotic is at four increasing concentrations, these microarrays could be used for evaluation of inhibition concentration (IC). The growth kinetics of tested bacteria as reaction to antibiotic sensitivity with inhibition concentration marked are presented in Fig. 3. The results obtained confirm that the PM approach may be used as an additional tool to indicate variations in antibiotic sensitivity of the environmental bacteria and in preliminary detection of inhibition concentrations.

**Table 1:** Antibiotic susceptibility profile of selected bacteria determined by PM11 and PM12 microarrays

	*Acinetobacter johnsonii*	*Aeromonas bestiarum*	*Alcaligenes faecalis*	*Flavobacterium hydatis*	*Microbacterium flavescens*	*Pseudomonas fluorescens*	*Serratia marcescens*	*Stenotrophomon-as maltophili*	*Stenotrophomon-as rhizophila*	*Variovorax paradoxus*
Aminoglycosides										
Amikacin	R	R	R	R	R	R	R	R	R	R
Neomycin	R	R	R	R	R	R	R	R	R	R
Gentamicin	R	R	R	R	R	R	R	R	R	R
Kanamycin	R	R	R	R	R	R	R	R	R	R
Paromomycin	S	R	R	R	S	S	S	R	R	R
Sisomicin	S	R	R	R	S	R	R	R	R	R
Novobiocin	S	S	S	S	S	R	S	R	R	R
Tobramycin	S	R	R	R	S	S	R	R	R	R
Spectinomycin	S	R	R	R	R	R	R	R	R	R
β-lactams										
Amoxicillin	R	R	R	R	S	R	R	R	R	R
Cloxacillin	R	R	R	S	S	R	R	R	R	R
Nafcillin	R	R	R	S	R	S	R	R	R	R
Cefazolin	R	R	R	R	R	R	R	R	R	R
Ceftriaxone	R	R	R	R	S	R	R	R	R	R
Cephalothin	R	R	R	R	R	R	R	R	R	R
Penicillin G	R	R	R	S	R	R	R	R	R	R
Carbenicillin	R	R	R	R	R	R	R	R	R	R
Oxacillin	S	R	R	S	S	R	R	R	R	R
Lincosamides										
Lincomycin	R	R	R	S	S	R	R	R	R	R
Synthetic antibiotics										
Lomefloxacin	S	R	R	R	R	R	S	R	R	R
Enoxacin	S	R	R	R	R	S	S	R	R	R
Nalidixic acid	S	R	S	S	S	R	S	R	R	R
Ofloxacin	S	R	S	R	R	R	R	R	R	R
Glycopeptides										
Bleomycin	S	R	R	R	S	R	R	R	R	R
Colistin	S	S	R	R	R	S	R	R	R	R
Capreomycin	S	R	R	R	R	R	R	R	R	R
Polymyxin B	S	S	R	R	R	R	R	R	R	R
Vancomycin	S	R	R	S	S	R	R	R	R	R
Tetracyclines										
Chlortetracycline	S	R	R	S	R	R	R	R	R	R
Minocycline	S	R	R	S	R	R	S	R	R	R
Demeclocycline	S	R	R	S	S	R	R	R	R	R
Tetracycline	S	R	R	R	S	R	R	R	R	R
Penimepicycline	S	R	R	S	S	R	R	R	R	R
Amphenicols										
Chloramphenicol	R	S	R	S	S	R	R	R	S	R
Macrolides										
Erythromycin	R	R	S	S	S	R	R	R	R	S
Spiramycin	R	S	S	S	S	S	R	R	R	R
Sulfonamides										
Sulfamethoxazole	S	R	R	R	R	R	R	R	R	R
Sulfathiazole	S	R	S	R	R	R	R	R	R	R
Sulfadiazine	S	R	S	R	R	R	R	R	R	R
Sulfamethazine	R	R	S	R	R	R	R	R	R	R
Rifamycins										
Rifampicin	S	S	R	S	S	R	R	S	R	R

*S* sensitive; *R* resistant

Recently, more attention has been focused on using PMs to direct high-throughput assessment of cellular phenotypes (phenome). Most of the papers screen the metabolic capabilities and chemical sensitivity of various bacteria (Biondi et al. 2009; Zhang et al. 2009; Line et al. 2010; Decorosi et al. 2011; Lucas and Manna 2013; Scaria et al. 2014). The information from the papers is useful to highlight modifications of metabolic properties of pathogens, pathogen-related bacteria, or bacteria with biotechnological potential use in bioindustry. The results presented by Scaria et al. (2014) give the comprehensive nutritional requirements and chemical sensitivity profile of six *Clostridium difficile* strains of varying virulence. These properties could be used for designing better interventions for the treatment of recurrent *C. difficile* infection and also for formulating tube feeding formulas that could reduce the infection risk.

In this paper, special attention is given to present the additional application of phenotype microarray as a modern tool for identification of antibiotic sensitivity of bacteria and for detection of antibiotics concentration to inhibit the growth of bacteria. The four increasing concentrations of each antibiotic were tested by PMs. However, knowledge on the variations of antibiotics concentrations in wastewater is needed for the further analysis of the results. Up to now, the antibiotic susceptibility is mostly performed by the disc diffusion method, microdilution procedure, or molecular approach. The advantages of PMs over the traditional methods like diffusion method are (1) more than 40 antibiotics belonging to ten different chemical classes can be analyzed simultaneously, (2) time and chemicals saving method, (3) much easier to perform, (4) simply preparing standardized cell suspension and inoculating the microwells, and (5) the results are read automatically. A part of this technology is OmniLog instrument which can automatically read and record the color change in PM assays. The instrument cycles microplates in front of a color CCD camera to read 50 microplates in as little as 5 min and provides quantitative and kinetic information on the response of the cells in the PMs. The data are stored directly into the computer files and can be recalled, analyzed, exported in a variety of raw and processed forms, generated reports, and compared with other data at any time. However, the method is only applicable to analyze the culturable fraction of bacteria.

In conclusion, small wastewater treatment plants may be contributed to the spread of antibiotic-resistant bacteria in the natural environment. Another issue addressed in this study was to note the possibility of using the Biolog’s microarrays for determining of resistant phenotypes of a culturable fraction of environmental bacteria. The results demonstrate the applicability of the microarrays to establish antibiotic susceptibility profiles of the environmental bacterial strains. Although further research is required, phenotype microarrays could be successfully used as a modern tool for identification of the multi-antibiotic resistance of bacteria and for preliminary establishing of the inhibition concentrations (ICs).
